# Crossing the Line: Seroprevalence and Risk Factors for Transboundary Animal Diseases Along the Tanzania-Zambia Border

**DOI:** 10.3389/fvets.2022.809128

**Published:** 2022-03-11

**Authors:** Sara Lysholm, Johanna F. Lindahl, Musso Munyeme, Gerald Misinzo, Coletha Mathew, Karin Alvåsen, George Dautu, Siri Linde, Lydia Mitternacht, Emelie Olovsson, Elsa Wilén, Mikael Berg, Jonas J. Wensman

**Affiliations:** ^1^Department of Clinical Sciences, Swedish University of Agricultural Sciences, Uppsala, Sweden; ^2^Department of Medical Biochemistry and Microbiology, Uppsala University, Uppsala, Sweden; ^3^Department of Biosciences, International Livestock Research Institute, Nairobi, Kenya; ^4^Department of Disease Control, School of Veterinary Medicine, University of Zambia, Lusaka, Zambia; ^5^SACIDS Foundation for One Health, Sokoine University of Agriculture, Morogoro, Tanzania; ^6^Department of Veterinary Anatomy and Pathology, Sokoine University of Agriculture, Morogoro, Tanzania; ^7^Department of Veterinary Services Ministry of Fisheries and Livestock, Central Veterinary Research Institute, Lusaka, Zambia; ^8^Department of Biomedical Science and Veterinary Public Health, Swedish University of Agricultural Sciences, Uppsala, Sweden; ^9^Department of Epidemiology and Disease Control, National Veterinary Institute, Uppsala, Sweden

**Keywords:** brucellosis, foot and mouth disease, peste des petits ruminants, Rift Valley fever, sheep and goat pox, seroprevalence, risk factors

## Abstract

Transboundary pathogens pose a threat to livelihood security in countries such as Zambia and Tanzania. This study aimed to investigate the seroprevalence of peste des petits ruminants virus (PPRV), foot and mouth disease virus (FMDV), sheep and goat pox virus (SGPV), Rift Valley fever virus (RVFV) and *Brucella* spp. in sheep and goats along the Tanzania-Zambia border. Another aim was to assess the association between certain predictor variables and seroprevalence, focusing on trade and proximity to an international border, to a town and to the Tanzania-Zambia highway. During September-October 2018, 486 serum samples from small ruminants in Zambia and 491 in Tanzania were collected and analyzed using enzyme-linked immunosorbent assays (ELISA). A questionnaire focused on management strategies was administered to each household. The animal-level seroprevalence in Zambia was 0.21% [95% confidence interval (CI) (0.01–1.14) for PPRV, 1.03% (95% CI 0.33–2.39) for FMDV, 0% (95% CI 0–0.76) for SGPV, 2.26% (95% CI 1.14–4.01) for RVFV and 1.65% (95% CI 0.71–3.22) for *Brucella* spp.]. In Tanzania, animal-level seroprevalence was 2.85% (95% CI 1.57–4.74) for PPRV, 16.9% (95% CI 13.7–20.5) for FMDV, 0.20% (95% CI 0.01–1.13) for SGPV, 3.26% (95% CI 1.87–5.24) for RVFV and 20.0% (95% CI 14.5–26.5) for *Brucella* spp. For PPRV (OR 6.83, 95% CI 1.37–34.0, *p* = 0.019) and FMDV (OR 5.68, 95% CI 1.58–20.3, *p* = 0.008), herds situated more than 30 km from an international border were more likely to be seropositive, while being located 10–30 km (OR 4.43, 95% CI 1.22–16.1 *p* = 0.024) from a border was identified as a risk factor for *Brucella* spp. For FMDV (OR 79.2, 95% CI 4.52–1388.9, *p* = 0.003), being situated within 30 km from a town was associated with seropositivity. Furthermore, contact with wild ruminants (OR 18.2, 95% CI 1.36–244), and the presence of sheep in the household (OR 5.20, 95% CI 1.00–26.9, *p* = 0.049), was associated with seropositivity for PPRV, and FMDV. No significant associations between trade or distance to the Tan-Zam highway and seroprevalence were found. We recommend that the impact of trade and proximity to borders, towns and roads should be further evaluated in larger studies, ideally incorporating aspects such as temporal trade fluctuations.

## Introduction

Small ruminants (sheep and goats) are increasingly being recognized for their role in securing the livelihoods of farmers, traders and other stakeholders, especially in low- and lower-middle-income countries. Sheep and goats are important for income generation and storing wealth, and for food and nutritional security (1). However, these roles are severely constrained by transboundary animal diseases (TADs), which are contagious and capable of rapid cross-regional spread, irrespective of international borders. These diseases can have severe socio-economic consequences, apart from their direct and immediate impacts on trade, food and nutritional security, and animal welfare (2). Tanzania and Zambia are neighboring countries in south-east Africa, and despite their proximity, there are considerable differences in the spectra of transboundary animal pathogens reported in each of the countries. Border porosity, a high level of cross-border small ruminant movements and trade, as well as poor biosecurity pose a substantial risk of pathogen exchange across the border between the two countries (3–5).

There are several TADs that affect small ruminants, such as peste des petits ruminants (PPR), foot and mouth disease (FMD), sheep- and goat pox (SGP), Rift Valley fever (RVF) and brucellosis. Peste des petits ruminants is a highly contagious disease caused by PPR virus (PPRV), a member of the morbillivirus genus. Clinical signs indicative of PPRV include pyrexia, mucopurulent oculonasal discharges, cough, dyspnea, necrotic stomatitis and severe diarrhea (6). Morbidity and case fatality rates can be as high as 100 and 90%, respectively, in naïve populations (7). Due to the severe impacts of PPR, the OIE and FAO joined forces in 2015 with the goal of eradicating PPRV by 2030 (7). While PPRV is endemic in Tanzania (8), antibodies against PPRV in small ruminants without proven presence of the virus or clinical disease have been reported in Zambia (9). However, the risk of PPRV introduction into Zambia from Tanzania is considered high (3, 4). The fact that PPRV has also been detected in the neighboring Democratic Republic of the Congo (DRC) (10–13) and Angola (14), further increases the risk of PPRV introduction into Zambia.

Foot and mouth disease, caused by FMD virus (FMDV), is endemic in both Zambia and Tanzania (15–19). Despite a relatively low mortality rate, FMD has been identified as the principal animal disease of global concern due to its high morbidity and severe negative effects on animal production and trade (2). Since sheep and goats often only develop mild and transient clinical signs that are easily missed, they can act as reservoirs and contribute to the spread of the virus, as was seen in the FMD outbreak in the UK in 2001 (20). The fact that small ruminants are often excluded from vaccination campaigns for FMDV (21) further increases their ability to act as reservoirs. Sheep- and goatpox (here abbreviated as SGP) is another TAD caused by two closely related capripoxviruses, i.e., sheeppox virus (SPPV) and goatpox virus (GTPV), or sheep- and goatpox virus (here called SGPV). Both viruses are generally host-specific, although cross-transmission does occur (22). Sheep- and goatpox virus can cause high morbidity and mortality rates in small ruminants, especially in exotic breeds that are imported into an endemic area. The disease is characterized by pyrexia, generalized skin and internal pox lesions and lymphadenopathy (23). Sheep- and goatpox virus has not been discovered in Zambia, but has been found in both neighboring Tanzania (24) and DRC (11).

There are several TADs of sheep and goats that are zoonotic and capable of causing disease in humans as well as small ruminants. Rift Valley fever, caused by RVF virus (RVFV), is generally manifested by periodic epizootic outbreaks of abortions and neonatal mortalities in sheep, goats and cattle, as well as influenza-like and hemorrhagic disease in humans (25). While the disease is mainly transmitted between animals by mosquito bites, humans are typically infected following contact with blood and offal at slaughter or when consuming undercooked meat (26). Rift Valley fever virus is endemic in both Zambia and Tanzania, although no outbreaks have been reported in Zambia since 1989 (27) or in Tanzania since 2006–07 (28, 29).

Brucellosis is caused by members of the bacterial genus *Brucella*, and the disease is endemic in both Zambia and Tanzania. Small ruminants are generally infected by *Brucella melitensis, B. abortus, B. suis* and *B. ovis* (30), of which all but *B. ovis* are zoonotic (31). Humans risk infection when they come into contact with aborted fetuses or fetal membranes, e.g., when assisting an animal during parturition, and through consumption of unpasteurized dairy products and undercooked meat, or when slaughtering an animal (31). Clinical signs indicative of infection with *Brucella* spp. in sheep and goats include abortions, stillbirths and occasionally joint hygromas, orchitis and epididymitis (30), while common symptoms in humans are undulant fever, weakness, general malaise and joint pain (31).

Increased participation in animal trade has the potential to improve the livelihoods of poor smallholder farmers (32, 33), but it can also contribute to the transmission of diseases, both TADs and endemic diseases (34, 35). Livestock trade and animal movement tend to occur at higher frequencies close to international borders, which in turn can contribute to increased exposure to transboundary animal pathogens. Clustering of outbreaks of FMD in cattle in both Zambia and Tanzania have previously been shown close to international borders, including the Tanzania-Zambia border region. Furthermore, clustering has been observed close to the Tanzania-Zambia (or Tan-Zam) highway, a major transportation route connecting the two countries (15–19).

The aim of this study was to assess the seroprevalence of PPRV, FMDV, SGPV, RVFV, and *Brucella* spp. in sheep and goats in the Tanzania-Zambia border region. The study also aimed to investigate associations between certain predictor variables and seroprevalence, focusing on trade and proximity to the Tanzania-Zambia or Zambia-Malawi borders, to a larger town and to the Tan-Zam highway.

## Materials and Methods

### Study Area and Study Design

**Figure 1:**
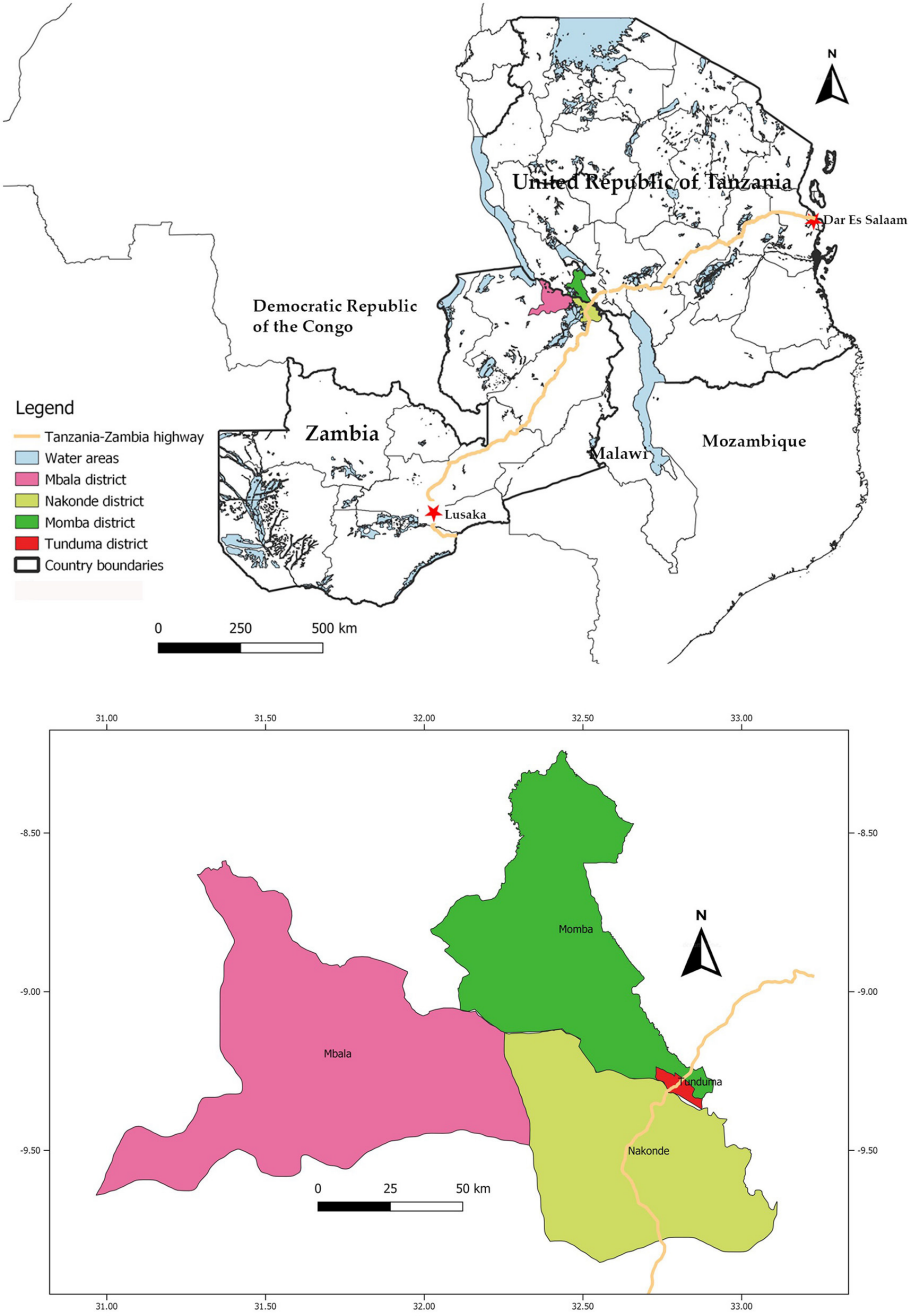
The location of the visited districts in the Tanzania-Zambia border region, and of the Tanzania-Zambia highway. Map created in QGIS version 3.4.4 software (https://qgis.org).

The study was designed to provide cross-sectional data on the seroprevalence of PPRV, FMDV, SGPV, RVFV and *Brucella* spp., and potential associations between selected predictor variables and seroprevalence, in sheep and goats in the border region between Zambia and Tanzania. Four districts, two in Zambia and two in Tanzania, where purposively selected for the study: Nakonde and Mbala districts in Zambia, and Tunduma and Momba districts in Tanzania. Mbala district was chosen due to its immediate proximity to the border, while Momba, Nakonde and Tunduma districts were chosen due to their border proximity and because the Tan-Zam highway runs through them (Figure 1). In Tanzania, there is a global and regional aligned national control program for PPR, and sero-surveys and vaccination campaigns have previously been conducted in both Tunduma and Momba districts. Unfortunately, these activities had to be scaled down in 2013 due to lack of funding (E. S. Swai, personal communication). Vaccinations for RVF were performed in Tunduma and Momba district until 2008, after which measures aiming to control the mosquito population were deemed more cost-efficient and therefore used instead (D. Mdetele, personal communication). Furthermore, both Zambia and Tanzania have control programs aiming to control the spread of FMD, although these efforts are mainly directed at cattle. There are no control programs for small ruminant diseases in Zambia.

Village lists were obtained from local veterinary personnel, from which 40 villages on each side of the border were randomly selected using the randomize tool in excel. The lists were later edited with the assistance of local veterinary personnel, and villages that were inaccessible or did not contain sheep or goats were replaced with a neighboring village. Due to difficulties in finding sufficient numbers of small ruminants in some villages, 41 villages ended up being included in Tanzania and 42 in Zambia. On the Zambian side, 32 villages in Nakonde district and 10 in Mbala district were visited. More villages were visited in Nakonde district due to the high frequency of cross-border trade and the fact that the Tan-Zam highway transects the district. In Tanzania, 33 villages were randomly selected in Momba district, as Tunduma district only consisted of eight villages. In each village, four households were selected using snowball sampling methodology (36). To diversify which farmers were approached, conditions based on herd size were included in the snowball sampling strategy. For example, the farmers were asked to re-direct us to a small ruminant farmer who fulfilled one of the following criteria: (i) a household that kept fewer than five sheep and/or goats, (ii) a household that kept 5–15 sheep and/or goats, and (iii) a household that kept more than 15 sheep and/or goats. These groups were based on the average herd size of sheep and goats in Zambia (8.4 and 7.4, respectively) and Tanzania (5.2 and 6.8, respectively) (37, 38). The goal was to include at least one household in each group per village. If all three groups were included with the first three households, no conditions were given for the last household. If it was not possible to find representatives of all groups, another household was included irrespective of the group to which it belonged. Samples were obtained from three sheep or goats per household. The selection was non-random as it was generally the farmer who chose the animals to be sampled. Animals younger than 4 months of age were excluded to avoid interference from maternal antibodies. In the cases where it was not possible to obtain samples from three animals in a given household, more samples were collected either from subsequent households or from households in other villages.

The study was conducted in two different strata, specifically the Tanzanian and the Zambian sides of the border. The sample size per stratum was calculated assuming an infinite population, and using a 95% confidence interval (CI), 5% margin of error, 50% assumed true prevalence, and sensitivity and specificity values from the ID Vet *ID Screen Rift Valley Fever Competition Multi-species* competitive enzyme-linked immunosorbent assay (cELISA), in order to generate the largest required sample size. The sample size calculation was performed according to the instructions in Humphry, Cameron (39), using the “Sample size to estimate a true prevalence with an imperfect test” tool on the https://epitools.ausvet.com.au/ webpage (Ausvet, Australia). The necessary sample size was calculated to be 461, which was subsequently rounded up to 480 sheep or goats to allow for errors in sample procurement and analysis.

### Sample and Data Collection

Data were collected between September and October 2018. Blood samples were obtained from the jugular vein using sterile needles and plain vacutainer tubes (BD Vacutainer, Plymouth, UK). The samples were then left standing in a cool box to coagulate and separate. At the end of each day, the serum was transferred to cryotubes and placed in a freezer at ~-20°C. The samples were later transported to an ultralow freezer for long-term storage at −80°C. For each sampled individual, information on age, sex, origin and signs of disease, both on the day of sample collection and within the last 12 months, was recorded. In addition, epidemiological data on herd characteristics and biosecurity, trade routines, management practices and herd disease history were collected in a questionnaire. The questionnaire was constructed using experience from interviews with farmers and veterinary personnel in Nakonde district and veterinary personnel in Tunduma district conducted by the first author in April-May 2018. The questionnaire was written in English and translated by an enumerator into the local languages, namely Swahili in Tanzania and Namwanga or Mambwe in Zambia. The enumerator asked the questions orally, clarified misunderstandings where necessary, and manually recorded the respondent's answers in English on the questionnaire sheet. The questionnaire took ~20–30 min to complete and contained a mix of open and closed questions.

Furthermore, the global positioning system (GPS) coordinates for each of the visited households were recorded. Distances to the Tanzania-Zambia and Zambia-Malawi borders, to the Tan-Zam highway and to a town were measured by entering the coordinates into ArcGIS online (Esri, California, USA), and using the distance measurement tool. Due to difficulties in obtaining data on road infrastructure in the study area, the closest linear distance was measured. When measuring border proximity, the linear distance to the closest border, either the Tanzania-Zambia or Zambia-Malawi border, was recorded. In most instances, the distance to the Tanzania-Zambia border was shorter, however eight households in Nakonde district in Zambia were closer to the Zambia-Malawi border and hence this distance was recorded. When measuring the distance to a town, Mbeya, Tunduma, Vwawa and Sumbawanga towns were included in Tanzania, and Nakonde and Mbala towns in Zambia.

### Laboratory Analysis

Laboratory analysis was performed using commercially available enzyme-linked immunosorbent assays (ELISA) to detect antibodies against the selected pathogens. The following kits were used: *ID Screen PPR competition ELISA* (sensitivity 100%, specificity 100%; ID Vet, Grabels, France), *ID Screen FMD NSP competition* (sensitivity 100%, specificity 99.5%; ID Vet, Grabels, France), *ID Screen Capripox Double Antigen Multi-species* which detects antibodies to SPPV, GTPV and lumpy skin disease virus (LSDV) (sensitivity not known, specificity 99.7% in capripox-free regions; ID Vet, Grabels, France) and *ID Screen Rift Valley fever Competition Multi-species* (sensitivity 100%, specificity 100%; ID Vet, Grabels, France). For *Brucella* spp., two different cELISA were used: in Zambia *Svanovir Brucella-Ab C-ELISA* (sensitivity 100%, specificity 100%; Boehringer-Ingelheim Svanova Diagnostics, Uppsala, Sweden), and in Tanzania LTELISA *Brucella* cELISA kit, (sensitivity 98.9%, specificity 99.9%; LT Biotech Moksklininku 6A, Vilnius, Lithuania). Both ELISAs detect antibodies to *B. abortus, B. melitensis* and *B. suis*. The *Brucella* spp. analysis in Tanzania was conducted more than a year after the other analyses, and at that time a large number of samples could no longer be located. Hence, the number of analyzed samples for *Brucella* spp. in Tanzania was lower (*n* = 671) compared with the other pathogens.

Independent evaluations of the sensitivity and specificity have been performed on the PPRV and RVFV ELISAs, and the *Brucella* spp. ELISA used in Zambia. For the PPRV ELISA, sensitivity and specificity was estimated to be 94.5 and 99.4%, respectively (40). For RVFV, one study estimated sensitivity and specificity to both be 100% (41), and another to be 91–100% and 100%, respectively (42). For the *Brucella* spp. ELISA that was used in Zambia, sensitivity and specificity values of 99.4 and 98.9%, respectively have been estimated (43). All the kits were utilized, validated and interpreted according to the instructions provided by the manufacturer. The results in the PPR and RVF ELISAs were scored as positive, negative or doubtful. Doubtful samples were considered negative in the statistical analysis.

### Statistical Analysis

**Figure 2:**
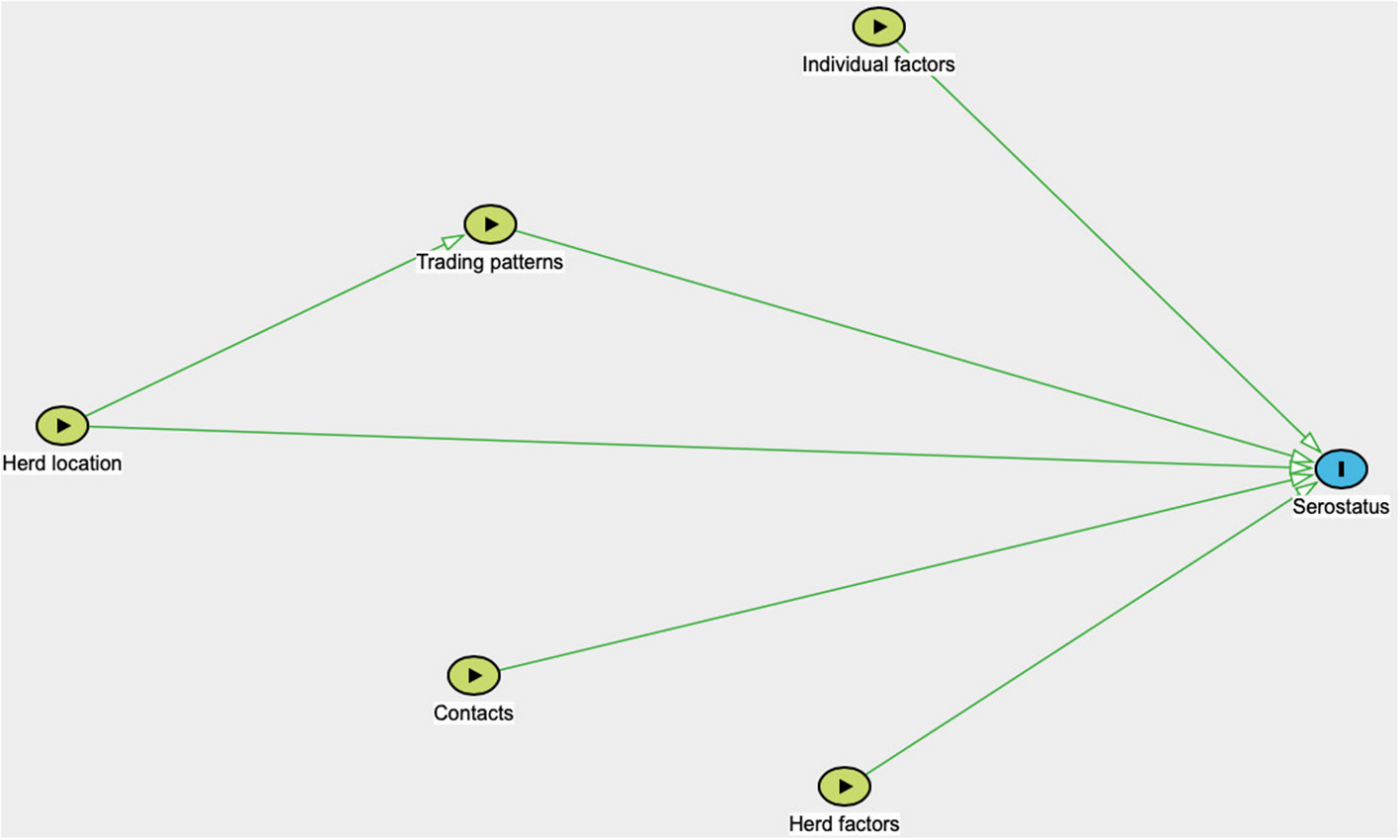
Directed acyclic graph illustrating variables potentially associated with pathogen seroprevalence. Herd location include the village-location, district-location and country-location of the sampled herd, as well as distance to the nearest international border, town and the Tan-Zam highway. Trading patterns include introduction of new animals, selling, buying from traders or at markets, and buying from or selling to other countries. Contact includes contact with wildlife and sheep, goats and cattle from other herds. Individual factors include age, sex, and species. Herd factors include herd size.

True prevalence was calculated using the apparent prevalence and the sensitivity and specificity of the statistical test, in accordance with Rogan and Gladen (44) and using the “Estimated true prevalence and predictive values from survey testing” on https://epitools.ausvet.com.au (Ausvet, Australia). Furthermore, statistical analyses were performed using Stata IC 16/1 (StataCorp LLC, USA). Univariable and multivariable analyses were conducted on both animal- and herd-level data for the respective pathogens. A herd was considered seropositive for a pathogen if one or more of the sampled animals in the herd tested positive. As the sampling strategy was conducted in a population where all villages in the end were not eligible for practical reasons, and the number of farming households per village were not known, no corrections for sampling weights were done, and prevalence presented is unadjusted. The potential predictive variables included were age, sex, species, country, treatment frequency with acaricides, time of latest introduction of a new small ruminant to the herd, whether the household has bought sheep and/or goats from traders or at markets, time of latest selling event of a small ruminant, purchase of sheep or goats from other countries or residing in a community where this is done, selling sheep or goats to other countries or residing in a community where this is done, contact frequency with sheep and goats from other herds, contact frequency with cattle from other herds, contact with wild ruminants, herd size, proximity to the Tanzania-Zambia or Zambia-Malawi border, proximity to a town, and proximity to the Tan-Zam highway. Univariable analysis was conducted using the Chi2 test or Fischer's exact test where applicable. Multivariable analyses were performed in STATA using the meqrlogit command, a method which does not provide a pseudo *R*^2^ value. In the animal-level analysis, district, village and herd were included as random effects, and in the herd-level analysis, district and village were included as random effects. All predictive variables with a *p*-value of 0.25 or less in the univariable analysis were included in the multivariable analysis, unless multicollinearity was detected. Multicollinearity was tested in all the models using variance inflation factor (VIF), and a cut-off value of 10 was used. For example, the analysis identified collinearity between country and grazing strategy, therefore grazing strategy was removed from the analysis. The analyses were guided by directed acyclic graphs (Figure 2), where distance to the Tanzania-Zambia or Zambia-Malawi border was identified as a confounding variable. The frequencies of buying and selling new goats, as well as proximity to an international border, to a town and to the Tan-Zam highway were always included in the initial models, as these were of special interest for the scope of the study. Initially, the full model was run, and the variable with the highest conjoined *p*-value using the Wald Test was removed in a stepwise backward elimination procedure, which was continued until one significant variable remained. Confounding was controlled for in each step, and a variable was judged to be a confounder when it affected the coefficient of other models with >20%. However, no confounder was identified. Selection of the best fitting model was subsequently performed using the Akaike information criteria (AIC). Residual plots were also examined visually according to Dohoo and Martin (45). A *p*-value of <0.05 was considered statistically significant, but higher *p*-values are also presented here to show potential associations between a variable and seropositivity.

### Ethical Considerations

The data collection was performed in collaboration with Tanzanian and Zambian district veterinary officers. Informed written consent was acquired prior to data collection. For farmers who were unable to sign the consent sheet, a thumbprint was obtained instead. The study received ethical approval from the International Livestock Research Institute's (ILRI) Institutional Research Ethics Committee (IREC) (ILRI-IREC2018-04).

## Results

### Descriptive Statistics

In total, 977 animals originating from 324 households were sampled in this study (Table 1). In Tanzania, 491 sheep and goats were sampled, which originated from 164 different households belonging to 41 villages. In Momba and Tunduma districts, 396 and 95 animals were sampled, respectively. In Zambia, 486 animals were sampled, originating from 160 different households in 42 villages. In Mbala and Nakonde districts, 119 and 367 animals were sampled, respectively. The majority of the sampled animals were goats (97%), female (79%) and between 1 and 3 years of age (56%). Almost all of them were local mixed breeds. Herd size ranged from 2 to 210 animals, although only eight herds contained 50 or more animals, and 24 herds 20 or more animals. The median herd size was 8. Around 95% of the sheep and goats grazed on communal grazing grounds for at least part of the year, and 88 and 62% respectively were estimated to be in contact with small ruminants and cattle from other herds at least on a monthly basis. Only 21% of the sampled sheep and goats displayed clinical signs of disease at the time of visit, and 44% were reported to have been sick in the previous 12 months. Commonly reported clinical signs included ocular and nasal discharge, coughing and diarrhea. Only seven households reported that they had vaccinated their small ruminants; all of these were located in Tanzania and had been vaccinated against contagious caprine pleuropneumonia (CCPP) during March to May 2018. Also, 88 and 82% of the farmers reported to never have treated their small ruminants with acaricides or anthelmintic drugs.

**Table 1:** Distribution of samples collected from sheep and goats in Zambia and Tanzania in September to October 2018.

**Variable**		**Number of individuals**	**Proportion of individuals (%)**	**Number of herds**	**Proportion of herds (%)**
Total		977	100	324	100
Country	Zambia	486	49.7	160	49.4
	Tanzania	491	50.3	164	50.6
District	Nakonde (Zambia)	367	37.6	120	37.0
	Mbala (Zambia)	119	12.2	40	12.4
	Momba (Tanzania)	396	40.5	132	40.7
	Tunduma (Tanzania)	95	9.7	32	9.9
Species	Sheep	27	2.8		
	Goats	950	97.2		
Sex	Female	769	78.8		
	Male	207	21.1		
	Sex unknown	1	0.1		
Age group	<1 year	165	16.9		
	1–3 years	547	56.0		
	>3 years	260	26.6		
	Age unknown	5	0.5		

The majority (78%) of farmers had not bought, bartered or in any other way acquired new sheep or goats in the previous 12 months, and 62% of these farmers reported only having acquired new animals once when they started keeping small ruminants. The most common source by far for acquiring new small ruminants was from other farmers in the village or nearby villages, but 7.9% reported having bought from traders or at markets at least once. Most of the farmers (46%) had sold sheep or goats within the previous 12 months, while 25% had never sold a sheep or a goat (Table 2).

**Table 2:** Trade routines and distances to the Tanzania-Zambia or Zambia-Malawi border, to a town and to the Tan-Zam highway.

**Variable**		**Number of herds**	**Proportion (%)**
Last time new sheep or goats were acquired to the herd	In the last year	71	21.9
	More than 1 year ago	252	77.8
	Unknown	1	0.3
Last time sheep or goats were sold	In the last year	150	46.3
	More than 1 year ago	93	28.7
	Has never sold	80	24.7
	Unknown	1	0.3
Buying sheep or goats from traders or at markets	Yes	25	7.7
	No	292	90.1
	Unknown	7	2.2
Buying sheep or goats from other countries	Yes	8	2.5
	No	315	97.2
	Unknown	1	0.3
Selling sheep or goats to other countries	Yes	17	5.2
	No	306	94.5
	Unknown	1	0.3
Distance to the Tanzania-Zambia or Zambia-Malawi border	≤ 10 km	142	43.8
	>10 km-30 km	90	27.8
	>30 km	92	28.4
Distance to the Tan-Zam highway	≤ 10 km	77	23.8
	>10 km-30 km	45	13.9
	>30 km	202	62.3
Distance to a town	≤ 30 km	117	36.1
	>30–60 km	120	37.0
	>60 km	87	26.9

### Seroprevalence

Only one goat tested positive for PPRV in Zambia, which equals to an apparent animal-level seroprevalence of 0.21% (95% 0.01–1.14), while 2.85% (95% CI 1.57–4.74) sheep and goats were seropositive in Tanzania. For FMDV, apparent animal-level seroprevalence in Zambia and Tanzania was 1.03% (95% CI 0.33–2.39) and 16.9% (95% CI 13.7–20.5), respectively, while for SGPV, only one goat in Tanzania tested positive, equaling to a seroprevalence of 0.20% (95% CI 0.01–1.13) in Tanzania and 0% (95% CI 0–0.76) in Zambia. For RVFV and *Brucella* spp., animal-level seroprevalence in Zambia was 2.26% (95% CI 1.14–4.01) and 1.65% (95% CI 0.71–3.22), respectively, while 3.26% (95% CI 1.87–5.24) and 20.0% (95% CI 14.5–26.5) sheep and goats were seropositive in Tanzania (Tables 3, 4).

**Table 3:** Apparent animal-level seroprevalence for PPRV, FMDV and SGPV.

		**PPRV**		**FMDV**		**SGPV**	
		**Positive (analyzed)**	**% Seroprevalence (95% CI)**	**Positive (analyzed)**	**% Seroprevalence (95% CI)**	**Positive (analyzed)**	**% Seroprevalence (95% CI)**
Total		15 (977)	1.54 (0.86–2.52)	88 (976)	9.02 (7.29–11.0)	1 (977)	0.10 (0.00–0.57)
Country	Zambia	1 (486)	0.21 (0.01–1.14)	5 (485)	1.03 (0.33–2.39)	0 (486)	0 (0–0.76)^†^
	Tanzania	14 (491)	2.85 (1.57–4.74)	83 (491)	16.9 (13.7–20.5)	1 (491)	0.20 (0.01–1.13)
District	Nakonde (Zm)	1 (367)	0.27 (0.01–1.51)	3 (366)	0.82 (0.17–2.38)	0 (367)	0 (0–1.00)^†^
	Mbala (Zm)	0 (119)	0 (0–3.05)^†^	2 (119)	1.68 (0.20–5.94)	0 (119)	0 (0–3.05)^†^
	Tunduma (Tz)	1 (95)	1.05 (0.03–5.73)	23 (95)	24.2 (16.0–34.1)	0 (95)	0 (0–3.81)^†^
	Momba (Tz)	13 (396)	3.28 (1.76–5.55)	60 (396)	15.2 (11.8–19.1)	1 (396)	0.25 (0.01–1.40)
Species	Goats	15 (950)	1.58 (0.86–2.59)	84 (949)	8.85 (7.12–10.8)	1 (950)	0.11 (0.00–0.59)
	Sheep	0 (27)	0 (0–12.8)^†^	4 (27)	14.8 (4.19–33.7)	0 (27)	0 (0–12.8)^†^
Sex	Female	14 (769)	1.82 (1.00–3.04)	77 (769)	10.0 (7.98–12.4)	1 (769)	0.13 (0.00–0.72)
	Male	1 (207)	0.48 (0.01–2.66)	11 (206)	5.34 (2.70–9.35)	0 (207)	0 (0–1.77)^†^
Age group	<1 year	0 (165)	0 (0–2.21)^†^	0 (165)	0 (0–2.21)^†^	0 (165)	0 (0–2.21)^†^
	1–3 years	3 (547)	0.55 (0.11–1.59)	42 (546)	7.69 (5.60–10.3)	0 (547)	0 (0–0.67)^†^
	>3 years	12 (260)	4.62 (2.41–7.92)	45 (260)	17.3 (12.9–22.5)	1 (260)	0.38 (0.01–2.12)

† *One-sided confidence interval (97.5%)*.

**Table 4:** Apparent animal-level seroprevalence for RVFV and *Brucella* spp.

		**RVFV**		***Brucella*** **spp**.	
		**Positive (analyzed)**	**% Seroprevalence (95% CI)**	**Positive (analyzed)**	**% Seroprevalence (95% CI)**
Total		27 (977)	2.76 (1.83–4.00)	45 (671)	6.71 (4.93–8.87)
Country	Zambia	11 (486)	2.26 (1.14–4.01)	8 (486)	1.65 (0.71–3.22)
	Tanzania	16 (491)	3.26 (1.87–5.24)	37 (185)	20.0 (14.5–26.5)
District	Nakonde (Zm)	11 (367)	3.00 (1.51–5.30)	6 (367)	1.63 (0.60–3.52)
	Mbala (Zm)	0 (119)	0 (0–3.05)^†^	2 (119)	1.68 (0.20–5.94)
	Tunduma (Tz)	2 (95)	2.11 (0.26–7.40)	16 (51)	31.4 (19.1–45.9)
	Momba (Tz)	14 (396)	3.54 (1.95–5.86)	21 (134)	15.7 (9.97–23.0)
Species	Goats	26 (950)	2.74 (1.80–3.98)	45 (653)	6.89 (5.07–9.11)
	Sheep	1 (27)	3.70 (0.09–19.0)	0 (18)	0 (0–18.5)^†^
Sex	Female	22 (769)	2.86 (1.80–4.30)	39 (518)	7.53 (5.41–10.1)
	Male	5 (207)	2.42 (0.79–5.55)	6 (152)	3.95 (1.46–8.39)
Age group	<1 year	3 (165)	1.82 (0.38–5.22)	7 (128)	5.47 (2.23–10.9)
	1–3 years	16 (547)	2.93 (1.69–4.71)	27 (362)	7.46 (4.97–10.7)
	>3 years	8 (260)	3.08 (1.34–5.97)	11 (177)	6.21 (3.14–10.8)

† *One-sided confidence interval (97.5%)*.

Apparent herd-level seroprevalence for PPRV was 0.63% (95% CI 0.02–3.43) in Zambia and 7.30% (95% CI 3.84–12.4) in Tanzania. For FMDV and SGPV, herd-level seroprevalence in Zambia was 3.14% (95% CI 1.03–7.19) and 0% (95% CI 0–2.28) respectively, while 33.5% (95% CI 26.4–41.3) and 0.61% (95% CI 0.02–3.35) of the herds were seropositive in Tanzania. For RVFV and *Brucella* spp., herd-level seroprevalence was 5.62% (95% CI 2.60–10.4) and 5.00% (95% CI 2.18–9.61), respectively, in Zambia, while the corresponding numbers were 9.15% (95% CI 5.21–14.6) and 38.1% (95% CI 26.1–51.2), respectively, in Tanzania (Tables 5, 6).

**Table 5:** Apparent herd-level seroprevalence for PPRV, FMDV, and SGPV.

		**PPRV**		**FMDV**		**SGPV**	
		**Positive (analyzed)**	**% Seroprevalence (95% CI)**	**Positive (analyzed)**	**% Seroprevalence (95% CI)**	**Positive (analyzed)**	**% Seroprevalence (95% CI)**
Total		13 (324)	4.01 (2.15–6.76)	60 (323)	18.6 (14.5–23.3)	1 (324)	0.31 (0.01–1.71)
Country	Zambia	1 (160)	0.63 (0.02–3.43)	5 (159)	3.14 (1.03–7.19)	0 (160)	0 (0–2.28)^†^
	Tanzania	12 (164)	7.30 (3.84–12.4)	55 (164)	33.5 (26.4–41.3)	1 (164)	0.61 (0.02–3.35)
District	Nakonde (Zm)	1 (120)	0.83 (0.02–4.56)	3 (119)	2.52 (0.52–7.19)	0 (120)	0 (0–3.03)^†^
	Mbala (Zm)	0 (40)	0 (0–8.81)^†^	2 (40)	5.00 (0.61–16.9)	0 (40)	0 (0–8.81)^†^
	Tunduma (Tz)	1 (32)	3.12 (0.08–16.2)	17 (32)	53.1 (34.7–70.9)	0 (32)	0 (0–10.9)^†^
	Momba (Tz)	11 (132)	8.33 (4.23–14.4)	38 (132)	28.8 (21.2–37.3)	1 (132)	0.76 (0.02–4.15)

† *One-sided confidence interval (97.5%)*.

**Table 6:** Apparent herd-level seroprevalence for RVFV and *Brucella* spp.

		**RVFV**		***Brucella*** **spp**.	
		**Positive (analyzed)**	**% Seroprevalence (95% CI)**	**Positive (analyzed)**	**% Seroprevalence (95% CI)**
Total		24 (324)	7.41 (4.80–10.8)	32 (223)	14.4 (10.0–19.6)
Country	Zambia	9 (160)	5.62 (2.60–10.4)	8 (160)	5.00 (2.18–9.61)
	Tanzania	15 (164)	9.15 (5.21–14.6)	24 (63)	38.1 (26.1–51.2)
District	Nakonde (Zm)	9 (120)	7.50 (3.49–13.8)	6 (120)	5.00 (1.86–10.6)
	Mbala (Zm)	0 (40)	0 (0–8.81)^†^	2 (40)	5.00 (0.61–16.9)
	Tunduma (Tz)	2 (32)	6.25 (0.77–20.8)	9 (18)	50.0 (26.0–74.0)
	Momba (Tz)	13 (132)	9.85 (5.35–16.3)	15 (45)	33.3 (20.0–49.0)

† *One-sided confidence interval (97.5%)*.

For FMDV, true unadjusted animal- and herd-level seroprevalence in Zambia were 0.53% (95% CI 0.00–1.90) and 2.66% (95% CI 0.85–6.68), respectively, while the corresponding numbers in Tanzania were 16.5% (95% CI 13.4–20.1) and 33.2% (95% CI 26.4–40.8), respectively. True prevalence could not be calculated for SGPV since the sensitivity of the utilized ELISA is not known. For PPRV and RVFV, true prevalence was the same as the apparent prevalence, since the manufacturers of the ELISA tests that were utilized report 100% sensitivity and specificity. However, if the sensitivity and specificity values found in independent studies were used, true individual-level and herd-level seroprevalence for PPRV in Tanzania was estimated to 2.40% (95% CI 1.18–4.40) and 7.15% (95% CI 3.87–12.5). In Zambia, true animal- and herd-level seroprevalence of PPRV could not be accurately calculated due to the low apparent seroprevalence. For RVFV, true unadjusted animal- and herd-level seroprevalence was 2.49% (95% CI 1.39–4.40) and 6.18% (95% CI 3.28–11.4), respectively, in Zambia, and 3.58% (95% CI 2.21–5.74) and 10.1% (95% CI 6.18–16.0), respectively, in Tanzania. For *Brucella* spp. in Zambia, true unadjusted herd-level seroprevalence using the sensitivity and specificity values detected in an independent study was estimated to be 3.97% (95% CI 1.48–8.60), while true animal-level seroprevalence could not be calculated due to the low apparent seroprevalence. True seroprevalence for *Brucella* spp. in Tanzania was estimated to be 20.1% (95% CI 15.0–26.6) at animal-level and 38.5% (95% CI 27.4–51.0) at herd-level (Tables 7, 8).

**Table 7:** True animal- and herd-level seroprevalence for PPRV and FMDV.

		**PPRV**		**FMDV**	
		**% Animal-level (95% CI)**	**% Herd-level (95% CI)**	**% Animal-level (95% CI)**	**% Herd-level (95% CI)**
Total		1.00 (0.35–2.04)	3.63 (1.87–6.54)	8.56 (6.91–10.5)	18.2 (14.3–22.8)
Country	Zambia	Not calculated	Not calculated	0.53 (0.00–1.90)	2.66 (0.85–6.68)
	Tanzania	2.40 (1.18–4.40)	7.15 (3.87–12.5)	16.5 (13.4–20.1)	33.2 (26.4–40.8)

*For PPRV, true seroprevalence could not be calculated in Zambia as the apparent seroprevalence was too low; For SGPV, true seroprevalence could not be calculated since the sensitivity of the utilized assay is unknown*.

**Table 8:** True animal- and herd-level seroprevalence for RVFV and *Brucella* spp.

		**RVFV**		***Brucella*** **spp**.	
		**% Animal-level (95% CI)**	**% Herd-level (95% CI)**	**% Animal-level (95% CI)**	**% Herd-level (95% CI)**
Total		3.04 (2.09–4.39)	8.14 (5.53–11.9)	Not calculated	Not calculated
Country	Zambia	2.49 (1.39–4.40)	6.18 (3.28–11.4)	Not calculated	3.97 (1.48–8.60)
	Tanzania	3.58 (2.21–5.74)	10.1 (6.18–16.0)	20.1 (15.0–26.6)	38.5 (27.4–51.0)

*For Brucella spp., a combined true seroprevalence in the border region as a whole could not be calculated as two different ELISA kits were used in each country. True animal-level seroprevalence in Zambia could not be calculated because of the low apparent seroprevalence*.

Approximately 88.3% of the animals and 69.1% of the herds were seronegative to all the tested pathogens. Furthermore, 5.22% of the animals and 8.64% of the herds were seropositive for two or more of the pathogens, and one animal tested positive for three pathogens, i.e., PPRV, FMDV, and RVFV. Animals that were seropositive for FMDV were significantly more likely to be seropositive for PPRV (OR 7.15, *p* < 0.001), RVFV (OR 7.79, *p* < 0.001) and *Brucella* spp. (OR 3.63, *p* = 0.008). Otherwise, no associations of seropositivity between different pathogens were found.

### Predictor Variable Analysis

Multivariable predictor variable analysis was performed for all the pathogens except SGPV, due to the low number of seropositive animals. For PPRV, the analysis was performed on the data from Tanzania since only one animal in Zambia tested positive. Predictor variables associated with herd-level seropositivity for PPRV are shown in Table 9, for FMDV in Table 10, for RVFV in Table 11, and for *Brucella* spp. in Table 12. Animal-level predictor variables are displayed in Supplementary Tables 1–4. Predictor variables with *p*-values <0.25 that were later included in the multivariable analysis, are found in Supplementary Tables 5–12. Unless otherwise specified, the results presented here were analyses made of herd-level data.

**Table 9:** Predictor variables associated with herd-level seropositivity for PPRV.

**Fixed herd-level variables**	**PPRV**				
		**Seroprevalence % (95% confidence interval)**	**Odds ratio (OR)**	**OR 95% confidence interval**	***p*-value**
Distance to the Tanzania-Zambia or Zambia-Malawi border	≤ 30 km	2.17 (0.26–7.63)	Baseline	Baseline	Baseline
	>30 km	13.9 (6.87–24.1)	6.83	1.37–34.0	**0.019**
Contact with wild ruminants	At least once a year	66.7 (9.43–99.2)	18.2	1.36–244	**0.028**
	More rarely or never	6.21 (3.02–11.1)	Baseline	Baseline	Baseline
When the household last sold sheep or goats	This year	9.59 (3.94–18.8)	2.72	0.73–10.2	0.137
	Has not sold within the last year	5.49 (1.81–12.4)	Baseline	Baseline	Baseline
Constant			0.01	0.00–0.07	<0.001
**Random effects parameters**			**Estimate**	**Std. Err**.	**95% Confidence interval**
	District		<0.001	<0.001	0
	Village		<0.001	<0.001	0

*p-values < 0.05 are in bold. The analysis was performed on data from Tanzania due to the low seroprevalence in Zambia. n = 164*.

**Table 10:** Predictor variables associated with herd-level seropositivity for FMDV.

**Fixed herd-level variables**	**FMDV**				
		**Seroprevalence % (95% confidence interval)**	**Odds ratio (OR)**	**OR 95% confidence** **interval**	**p-value**
Country	Zambia	3.14 (1.03–7.19)	Baseline	Baseline	Baseline
	Tanzania	33.5 (26.4–41.3)	20.7	3.38–127	**0.001**
Presence of sheep in the household	Yes	37.5 (15.2–64.6)	5.20	1.00–26.9	**0.049**
	No	17.6 (13.5–22.3)	Baseline	Baseline	Baseline
Contact with cattle from other herds	At least once a month	28.4 (22.2–35.1)	2.86	0.42–19.3	0.281
	More rarely or never	1.68 (0.20–5.94)	Baseline	Baseline	Baseline
Buying sheep and goats from traders or at markets	Yes	48.0 (27.8–68.7)	2.47	0.95–6.43	0.065
	No	15.8 (11.8–20.5)	Baseline	Baseline	Baseline
Distance to the Tanzania-Zambia or Zambia-Malawi border	≤ 10 km	9.86 (5.50–16.0)	Baseline	Baseline	Baseline
	>10–30 km	20.2 (12.4–30.1)	2.01	0.66–6.14	0.221
	>30 km	30.4 (21.3–40.9)	5.68	1.58–20.3	**0.008**
Distance to the TanZam highway	≤ 10 km	10.4 (4.59–19.4)	Baseline	Baseline	Baseline
	>10–30 km	28.9 (16.4–44.3)	1.44	0.33–6.33	0.626
	>30 km	19.4 (14.2–25.6)	11.8	0.93–150	0.057
Distance to a town	≤ 30 km	18.8 (12.2–27.1)	79.2	4.52–1389	**0.003**
	>30–60 km	13.5 (7.88–20.9)	2.13	0.80–5.70	0.132
	>60 km	25.3 (16.6–35.7)	Baseline	Baseline	Baseline
Constant			<0.01	0.00–0.01	<0.001
**Random effects parameters**			**Estimate**	**Std. Err**.	**95% confidence interval**
	District		<0.001	<0.001	0
	Village		<0.001	<0.001	0

*p-values < 0.05 are in bold. n = 313*.

**Table 11:** Predictor variables associated with herd-level seropositivity for RVFV.

	**RVFV**				
**Fixed herd-level variables**		**Seroprevalence % (95% confidence interval)**	**Odds ratio (OR)**	**OR 95% confidence interval**	***p*-value**
Buying sheep and goats from traders or at markets	Yes	16.0 (4.54–36.1)	1.98	0.58–6.74	0.275
	No	6.85 (4.23–10.4)	Baseline	Baseline	Baseline
Presence of sheep in the household	Yes	18.8 (4.05–45.6)	3.11	0.78–12.4	0.107
	No	6.82 (4.27–10.2)	Baseline	Baseline	Baseline
Distance to the Tanzania-Zambia or Zambia-Malawi border	≤ 10 km	4.23 (1.57–8.97)	Baseline	Baseline	Baseline
	>10–30 km	8.89 (3.92–16.8)	2.36	0.78–7.12	0.128
	>30 km	10.9 (5.34–19.1)	2.54	0.86–7.53	0.093
Constant			0.04	0.02–0.09	<0.001
**Random effects parameters**			**Estimate**	**Std. Err**.	**95% Confidence interval**
	District		<0.001	<0.001	0
	Village		<0.001	<0.001	0

*p-values < 0.05 are in bold. n = 317*.

**Table 12:** Predictor variables associated with herd-level seropositivity for *Brucella* spp.

**Fixed herd-level variables**	***Brucella*** **spp**.				
		**Seroprevalence % (95% confidence interval)**	**Odds ratio (OR)**	**OR 95% confidence interval**	***p*-value**
Country	Zambia	5.00 (2.18–9.61)	Baseline	Baseline	Baseline
	Tanzania	38.1 (26.1–51.2)	17.7	6.19–50.4	**<0.001**
Having introduced new sheep or goats to the herd	This year	6.00 (1.25–16.5)	Baseline	Baseline	Baseline
	More than 1 year ago	16.9 (11.6–23.3)	3.72	0.96–14.4	0.057
Presence of community members selling small ruminants to other countries	Yes	5.88 (0.72–19.7)	Baseline	Baseline	Baseline
	No	15.4 (10.6–21.4)	2.53	0.47–13.7	0.283
Distance to the Tanzania-Zambia or Zambia-Malawi border	≤ 10 km	9.91 (5.05–17.0)	4.05	0.94–17.4	0.060
	>10–30 km	23.6 (14.4–35.1)	4.43	1.22–16.1	**0.024**
	>30 km	10.0 (2.79–23.7)	Baseline	Baseline	Baseline
Constant			<0.01	0.00–0.03	<0.001
**Random effects parameters**			**Estimate**	**Std. Err**.	**95% Confidence interval**
	District		<0.001	<0.001	0
	Village		<0.001	<0.001	0

*p-values < 0.05 are in bold. n = 221*.

For PPRV, all seropositive animals were goats, females and originated from herds in daily contact with small ruminants and cattle from other herds. In the herd-level analysis, being situated more than 30 km from an international border was identified as a risk factor (OR 6.83, 95% CI 1.37–34.0, *p* = 0.019), and in fact no seropositive animals were found within a 10 km radius. Contact with wild ruminants (OR 18.2, 95% CI 1.36–244, *p* = 0.028) was identified as a risk factor, and in the animal-level analysis, being older than 3 years was associated with increased seropositivity (OR 82.0, 95% CI 2.87–2342, *p* = 0.010). No animals under 1 year of age tested positive for PPRV antibodies, which could indicate limited circulation of the virus in the 12 months leading up to the time of sampling in the surveyed areas.

In the herd-level analysis for FMDV, sheep and goats in Tanzania were significantly more likely to be seropositive than those in Zambia (OR 20.7, 95% CI 3.38–127, *p* = 0.001). Households that bought sheep and goats from traders or at markets were also more likely to be seropositive, although this association was not statistically significant (OR 2.47, 95% CI 0.95–6.43, *p* = 0.065). Furthermore, being situated within 30 km of a town (OR 79.2, 95% CI 4.52–1389, *p* = 0.003) and the Tanzania-Zambia/Zambia-Malawi border (OR 5.68, 95% CI 1.58–20.3, *p* = 0.008) was associated with a higher risk of seropositivity. The same was seen for households located more than 30 km of the Tan-Zam highway (OR 11.8, 95% CI 0.93–150.0, *p* = 0.057), although this was not statistically significant. Furthermore, an association between being seropositive and the presence of sheep in the herd, either solely or in addition to goats, was identified in the analysis (OR 5.20, 95% CI 1.00–26.9, *p* = 0.049). In the animal-level analysis, an association between seropositivity and a herd size of 21 or more animals was identified (OR 4.75, 95% CI 1.52–14.8, *p* = 0.007), and animals older than 3 years of age were significantly more likely to be seropositive (OR 5.03, 95% CI 2.46–10.3, *p* < 0.001). In fact, no animals under 1 year of age tested positive for antibodies to FMDV, indicating limited circulation of virus in the 12 months up to the time of sampling in the surveyed area.

For RVFV, a non-significant association was observed in the herd-level analysis between keeping sheep in the household and seropositivity (OR 3.11, 95% CI 0.78–12.4, *p* = 0.107). Furthermore, a non-significant association between the herd being situated more than 30 km from the Tanzania-Zambia or Zambia-Malawi borders and increased seroprevalence was identified (OR 2.54, 95% CI 0.86–7.53, *p* = 0.093). The same non-significant trends were seen in the animal-level analysis.

All animals seropositive for *Brucella* spp. in this study were goats. The herd-level analysis revealed that small ruminants in Tanzania were significantly more likely to be seropositive than those in Zambia (OR 17.7, 95% CI 6.19–50.4, *p* < 0.001). However, as two different ELISA kits were used, any comparisons should be made with caution. Interestingly, herds where no new animals had been introduced within the previous 12 months were seropositive to a higher extent, although this association was non-significant (OR 3.72, 95% CI 0.96–14.4, *p* = 0.057). As opposed to the other pathogens, the seroprevalence was higher within a 10 km distance (OR 4.05, 95% CI 0.94–17.4, *p* = 0.060), and significantly higher within a 10–30 km distance (OR 4.43, 95% CI 1.22–16.1, *p* = 0.024) from the Tanzania-Zambia or Zambia-Malawi international borders. In the animal-level analysis, a significant association was observed between seropositivity and being situated within 30 km of a town (OR 6.04, 95% CI 1.03–35.5, *p* = 0.047).

### Clinical Signs

The study found associations between herd seropositivity for PPRV and mortalities in lambs and/or kids (*p* = 0.020) and in adult sheep and/or goats (*p* = 0.001) in the herd during year leading up to the study. Also, abortions were significantly less likely to have occurred during the previous 12 months in herds that were seropositive for *Brucella* spp. (*p* = 0.022).

Furthermore, an association between FMDV seropositivity and the presence of ocular and nasal discharge at the time of sampling (*p* = 0.005) was found. The study also detected multiple associations between seroprevalence and observations of clinical signs in the individual animal by the farmer during the 12 months leading up to the study (Table 13). Associations were found between PPRV and coughing (*p* = 0.019) and diarrhea (*p* = 0.001, FMDV and coughing (*p* = 0.001) and diarrhea (*p* = 0.001), and between RVFV and ocular and nasal discharge (*p* = 0.001).

**Table 13:** Statistically significant associations between clinical signs observed in the individual animal during the year leading up to sample collection, and pathogen seropositivity.

**Clinical sign**	**Pathogen**	**Increased (I) or decreased (D) likelihood of seropositivity**	***P*-value**
Ocular and nasal discharge	RVFV	I	0.001
Coughing	PPRV	I	0.010
Coughing	FMDV	I	0.001
Diarrhea	PPRV	I	<0.001
Diarrhea	FMDV	I	0.001

## Discussion

This study investigated animal- and herd-level seroprevalence of the transboundary pathogens PPRV, FMDV, SGPV, RVFV and *Brucella* spp. along the Tanzania-Zambia border. In addition, these results were used to investigate associations between different predictor variables and seroprevalence, focusing on, but not limited to, trade routines and proximity to a town, to the Tan-Zam highway and to the Tanzania-Zambia or Zambia-Malawi borders.

In this study, the detected animal- and herd-level seroprevalence of PPR in Tanzania was 2.85 and 7.30%, respectively, and seropositive animals were found in both Tunduma and Momba districts. The detected seroprevalence was lower than that reported in previous studies, where animal-level seroprevalence has ranged from 13 to 47% (24, 46–52). Potential reasons for this include differences in surveyed years, study design, and study area, as most previous studies were conducted in the northern and central regions of the country. All seropositive animals in this study were goats, although the difference in seroprevalence between species was not statistically significant. While some previous studies in Tanzania did not detect significant differences in PPR seroprevalence in sheep vs. goats (46, 52), others have found higher seroprevalence in goats (50), which can be explained by a higher susceptibility to PPRV infection compared to sheep (53). At the same time, goats are considered more likely to develop severe disease and die following PPRV infection (53), which may explain why e.g., a study in Pakistan detected a significantly higher seroprevalence in sheep (54). PPR vaccination campaigns have been performed previously in both Momba and Tunduma district previously, but these activities were scaled down in 2013 (E.S. Swai, personal communication), i.e., 5 years before the samples for this study were collected. Nine of the 14 seropositive animals in this study were reported to be 5 years or older by the farmer. As the vaccine generally gives rise to life-long immunity (55), it cannot be excluded that some of the seroconversions observed are due to vaccination and not natural infection. In Zambia, only one two-year old male goat tested positive for antibodies to PPRV. The positive animal had been born into the herd and had not been vaccinated for any disease, and none of the four other sampled animals from the same herd were seropositive. This result was in line with the fact that only antibodies without connection to clinical disease have previously been found in Zambia (9). The seropositive goat in this study tested positive on two separate ELISA runs, but the result needs to be verified using another methodology, preferably the virus neutralization test (VNT) or its equivalent.

The seroprevalence of FMDV was significantly higher in Tanzania than in Zambia. In Tanzania, animal-level and herd-level seroprevalence was 16.9 and 33.5%, respectively, and seropositive animals were found in both Tunduma and Momba districts and in 31 different villages. Twenty-one of these villages contained two or more seropositive small ruminants. The detected seroprevalence is lower compared to a previous study conducted in another part of Tanzania, where animal-level seroprevalence of FMDV in small ruminants was 39% (46). The animal-level and herd-level seroprevalence in Zambia was 1.03 and 3.14%, respectively, and seropositive animals were found in both Nakonde and Mbala districts. While previous studies on FMD in Zambia have focused on cattle, the result is similar to findings in a study in Zimbabwe (56), where FMDV seroprevalence in goats was 1.5%. As the area along the Tanzania-Zambia border has repeatedly been identified as a hotspot for FMDV outbreaks in cattle (15–18), the detected seroprevalence in this study was low, especially on the Zambian side of the border. As mortality in adult animals resulting from FMDV infection is low (57), a higher seroprevalence would be expected in a hotspot area, especially since contact rates between the sampled herds and cattle were reported to be high. However, possible explanations for the low seroprevalence are the fact that the aerosol production of FMDV in small ruminants is relatively small (58) and their population turnover rate is high compared with cattle (59). Hence, the present findings do not exclude the potential of small ruminants playing a role in the epidemiology of FMDV in the region.

SGPV has yet to be detected in Zambia, while its presence has been confirmed in Tanzania (24) and the DRC (11). In this study, only one animal tested positive for the presence of antibodies to SGPV, a 2-year old female goat in Momba district in Tanzania, corresponding to an animal- and herd-level seroprevalence of 0.20 and 0.61%, respectively. As SGPV is a highly contagious disease and none of the other two animals in the herd tested positive, the possibility of this test being a false positive should not be excluded.

The animal-level and herd-level seroprevalence of RVFV in this study was 2.26% and 5.62% in Zambia, and 3.26 and 9.15% in Tanzania. As no RVFV outbreak has been reported in Zambia since 1989 (27) or in Tanzania since 2007 (28, 29), these results may indicate inter-epidemic circulation of RVFV in the area, something that has previously been found in both Zambia (60, 61) and Tanzania (62, 63). This result is similar to findings in previous studies conducted during inter-epizootic periods in Tanzania, where animal-level seroprevalence in sheep and goats ranged from 5.4 to 11.7% (62–64). RVF vaccinations were performed in Tunduma and Momba district until 2008, i.e., 10 years prior to this study. As none of the RVFV seropositive animals in Tanzania in this study were older than 8 years, it is unlikely that they had seroconverted due to vaccinations rather than natural infection.

In this study, detected animal- and herd-level seroprevalence of *Brucella* spp. was 1.65 and 5.00%, respectively, in Zambia, and 20.0 and 38.1%, respectively, in Tanzania, and antibodies to the bacteria were detected in all the surveyed districts. In Zambia, the results are in line with a previous study (65), while it is lower than the seropositivity rate detected in sheep and goats at two informal small livestock markets in Zambia (66). The detected seroprevalence in Tanzania was higher than that previously reported (67–70), which at least in part is likely to be due to differences in study design and laboratory tests used. All seropositive animals in this study were goats, and in Tanzania, several previous studies have detected higher seroprevalence in this species compared to sheep (67, 70).

In this study, herds seropositive for FMDV and PPRV were significantly more likely to be situated more than 30 km from an international border compared to herds located at closer proximity. The same tendency was observed for RVFV although the association was non-significant. These findings stand in contrasts to results from previous spatio-temporal studies (15–18), where reported outbreaks of FMD in cattle in Zambia and Tanzania have been shown to be clustered close to several international borders, including the Tanzania-Zambia border. However, all the participating households in the present study were situated within a 90 km radius of a border. In a spatial analysis, an outbreak of a disease in any of these households would probably have been identified as having occurred in proximity to an international border. For *Brucella* spp., on the other hand, the seroprevalence was higher in herds located at closer proximity to a border. Unfortunately, not all samples in Tanzania could be analyzed for the presence of antibodies to *Brucella* spp., and among the herds that were situated more than 30 km from the border, only 43% were included. As disproportionately few herds in this group were included in the analysis, this result needs to be interpreted with caution. In addition to the importance of border proximity, clustering of FMD outbreaks in cattle along the Tan-Zam highway have been identified (15, 16). One probable reason is that cattle are often moved along this road to markets or slaughterhouses in other parts of Zambia and/or Tanzania. In this study, however, seroprevalence of FMD in sheep and goats was found to be higher in households situated 30 km or more from this major infrastructural route, although this association was non-significant. Compared with cattle, small ruminants are often sold locally (71), making less use of major transport routes such as the Tan-Zam highway, thereby possibly reducing (but not eliminating) its epidemiological significance. It should also be noted that most of the participating households were situated more than 30 km from the highway and therefore, this result should be interpreted with caution.

Furthermore, a significant association between proximity to a town and seropositivity for FMDV was identified based on herd-level data, and for *Brucella* spp. based on animal-level data. In the Tanzania-Zambia border region, ‘goat soup' (or supu ya mbuzi in Kiswahili) is considered a delicacy and is often sold at local restaurants. Therefore, many farmers opt to trek or transport their goats to a nearby town to sell them to a restaurant owner, which may explain the observed association with FMDV seropositivity. As many restaurants have limited access to refrigerators and freezers, the goats will be kept alive until their meat is needed, possibly encountering local animals in communal pastures, enabling them to exchange infectious pathogens. Furthermore, cities are more likely to contain livestock markets, and proximity to these markets has been associated with seropositivity for FMDV in cattle in Ethiopia in a previous study (72).

The introduction of new sheep or goats to the herd (through other means than within-herd births of lambs and kids) within the previous 12 months was identified as a non-significant protective factor for *Brucella* spp. in this study. This result contradicts previous studies, where frequent purchase of new animals has been identified as a risk factor for brucellosis (73, 74) and FMD (75). In the present study, acquiring new animals for the herd was rare, and those who did mostly got them from other farmers in the village or from nearby villages. The fact that most sheep and goats grazed on communal grazing grounds and were in frequent contact with sheep and goats from other herds, i.e., regularly coming into contact with the potential pool of source animals from which the farmer would be buying, could explain why this variable generally was not identified as a risk factor in this study. For *Brucella* spp., one potential explanation for the negative association between having introduced small ruminants to the herd within the past year and seropositivity is differences in economic status and access to capital. However, this needs to be investigated in future studies.

In addition, a non-significant association between seropositivity for FMDV and buying sheep and goats at markets or from traders was identified. At small livestock markets, animals of different species are often kept in proximity under stressful conditions, which facilitates pathogen exposure and spread (76). Livestock markets have previously been associated with the spread of infectious diseases (34, 77), and as traders often bring previously purchased animals with them when moving between villages, thereby allowing them to come into contact with local animals, traders risk contributing to the spread of disease (78).

Interestingly, annual contact with wild ruminants was identified as a risk factor for PPRV. In addition to sheep and goats, which are the main hosts of the virus, antibodies have been detected in various species of wild ruminants (79–81). In a previous study in Tanzania, a potential spillover of PPRV from domestic to wild ruminants has been observed (82), and the possibility of transmission in the opposite direction should not be ruled out. The importance of wildlife in the epidemiology of PPRV is currently being investigated due to its relevance in the planned eradication of the virus (7, 83). Furthermore, an association between keeping sheep, either solely or in addition to goats, and seropositivity for FMDV and RVFV were found, although for RVFV, this finding was not statistically significant. Previous studies in Ethiopia have identified that keeping cattle and small ruminants together is a risk factor associated with FMDV seroprevalence in cattle (72, 84). A plausible explanation for this observation could be the mild clinical picture in sheep and goats, which enables them to act as silent spreaders to cattle (57). However, some studies also indicate that sheep (85) and goats (86) are less efficient transmitters of FMDV compared to e.g., cattle, and there are indications that outbreaks in sheep often are self-cleared due to poor transmissibility (58). For RVFV, sheep are generally considered more likely than goats and cattle to develop severe disease (87), seroprevalence is generally higher in sheep (88), and there are indications that this species are more likely to be exposed to RVF during outbreaks (89). However, to the authors' knowledge, there is currently no data indicating that sheep are more infectious, which in turn could lead to a higher seroprevalence in other species in mixed herds. Furthermore, associations on animal-level data were found between seropositivity for FMDV, and ages above 3 years and herd sizes of 21 animals or more. These findings were expected, since older animals have had more time to be exposed to pathogens (72) and since the infectious pressure often is increased in herds that contain many animals (90).

For FMDV and *Brucella* spp., the seroprevalence was significantly higher in Tanzania than in Zambia, although for *Brucella* spp. this difference should be interpreted with caution as two different ELISA kits were used. For PPRV, the seroprevalence was significantly higher in Tanzania in univariable analysis, while only data from Tanzania were included in the multivariable analysis as only one animal was seropositive in Zambia. Given the absence of a natural demarcation between the two countries and the high levels of cross-border animal movement and trade, it was expected that the difference in seroprevalence would be less pronounced. One possible explanation for the higher seroprevalence in Tanzania of FMDV, *Brucella* spp. and PPRV is that at the time of sample procurement, trade in small ruminants was mainly directed from Zambia to Tanzania due to the high demand for goat meat on the Tanzanian side, coupled with comparatively low prices in Zambia. Furthermore, the animals that were sampled in Tanzania in this study were significantly older than the ones in Zambia (*p* < 0.001) and were in contact more often with sheep and goats (*p* < 0.001) and cattle (*p* < 0.001) from other herds. Herd sizes in Tanzania were also significantly larger than in Zambia (*p* = 0.020).

In addition, the study detected associations between various clinical signs reported by the farmers and seropositivity for certain pathogens. Most of these were expected, however one interesting finding was that abortions were significantly less likely to have occurred in herds that were seropositive for *Brucella* spp. While abortion in pregnant females is one of the most important sequelae to infection with *Brucella* spp., most sheep and goats will only abort once in their lifetime (30). It is possible that the seropositive females were infected, aborted and seroconverted more than a year before the samples were collected. Also, other aspects related to reproductive performance such as infertility and fetal loss in early pregnancy, which are easily overlooked by the farmer, were not included in this study.

While this study generated interesting results, it has some limitations. The lack of a registry of the populations of small ruminant farmers and of sheep and goats in both Zambia and Tanzania, and of disease prevalence in different regions, makes it challenging to design and execute a completely randomized study and to calculate sampling weights for adjustment of estimates. As the diseases included in this study are contagious, it can be expected for positive cases to be clustered in herds and villages. The sample size was however calculated based on a simple random sample, and hence did not take aspects such as clustering into account. In an attempt to compensate for this, the sample size calculation assumed a 50% true prevalence and used the sensitivity and specificity values from the ELISA with the lowest values, to yield a large necessary sample size. Also, to reduce potential clustering effects, we opted to collect the samples in a relatively large number of villages and households, a choice that was based on the combined knowledge and experience of the author group. Ideally, however, the study should have been designed as a multi-staged cluster sampling (91), with a listing of households with different numbers of animals. Also, differences in herd size were not considered when estimating seroprevalence, and while variations in size in general were small among the surveyed herds, with 50% having <8 animals, taking these differences into account should ideally have been done. The fact that the only three animals were sampled also in large herds, may have introduced a bias toward the null, and a lower prevalence is likely reported for these farms than may be true. Furthermore, to reduce the inherent biases associated with snowball sampling, such as a higher likelihood of e.g., respondents with wide social networks to be included in research, an attempt was made to stratify the included households based on their herd size. This stratification was based on the average herd sizes of sheep and goats in Zambia and Tanzania, but as there are large variations in herd sizes in both countries (38, 92), average herd size is probably a poor indicator of the number of animals kept by the average Zambian or Tanzanian farmer. In addition, the fact that it generally was the farmer who chose which animals were sampled is a potential source of bias. While this selection process appeared random, it is possible that some farmers chose his or her healthiest animals in order to present him- or herself as a good farmer, while others may have selected their sickest animals in a desire to find out why they are not doing well. Furthermore, although relatively uncommon, there are pastoralist households in the region where the study was conducted (93) and a few of them participated in the study. Comparing the seroprevalence between pastoralist herds and mixed crop-livestock herds would have added interesting information, but unfortunately this aspect was not included. Lastly, this study used measurements of linear distance to e.g., towns and borders, but a more representative measurement would be distance by roads. Unfortunately, accurate infrastructural information for the region, which in addition to major roads also includes smaller, more informal routes, was not included or in many instances was not available. As small ruminants are often sold in the village or in a nearby town, these smaller routes can play an important role in disease epidemiology.

## Conclusions

This study indicates that FMDV, RVFV, and *Brucella* spp. are circulating in the Tanzania-Zambia border region, while SGPV did not appear to be widely present. The low seroprevalence of PPRV in Zambia implies that exposure to the virus is uncommon. However, its continued presence in Tanzania, together with the high degree of cross-border livestock movement, warrants continuous surveillance for PPRV in the area. The associations between seroprevalence and trade routines and proximity to a border, a town or the Tan-Zam highway varied in this study. It is recommended that these aspects should be investigated further in larger studies in future. If possible, seroepidemiological data should be combined with spatio-temporal analyses, ideally incorporating aspects such as the locations of small ruminant markets, informal livestock movement routes and temporal trade fluctuations. This has the potential to reduce the impact of inherent biases in spatio-temporal studies, e.g., the in general poor willingness among resource-constrained farmers to report disease and the lack of resources among veterinary personnel to confirm diagnoses (94, 95).

## Data Availability Statement

The raw data supporting the conclusions of this article will be made available by the authors, without undue reservation.

## Ethics Statement

The animal study was reviewed and approved by the International Livestock Research Institute's (ILRI) Institutional Research Ethics Committee (IREC) (ILRI-IREC2018-04). Written informed consent was obtained from the owners for the participation of their animals in this study.

## Author Contributions

SLy, JL, MM, GM, KA, GD, MB, and JW: conceptualization. SLy, CM, SLi, LM, EO, and EW: investigation. SLy, SLi, LM, EO, and EW: data curation. SLy and JL: formal analysis. SLy, MM, GM, and JW: project administration. SLy: writing—original draft. JL, MM, GM, CM, KA, GD, SLi, LM, EO, EW, MB, and JW: writing—review and editing. JL, MM, GM, MB, and JW: supervision. MM and JW: funding acquisition. All authors contributed to the article and approved the submitted version.

## Funding

This study was funded by the Swedish Research Council (Grant Nos. 2018-03956, 2016-05667, and 348-2014-4293).

## Conflict of Interest

The authors declare that the research was conducted in the absence of any commercial or financial relationships that could be construed as a potential conflict of interest.

## Publisher's Note

All claims expressed in this article are solely those of the authors and do not necessarily represent those of their affiliated organizations, or those of the publisher, the editors and the reviewers. Any product that may be evaluated in this article, or claim that may be made by its manufacturer, is not guaranteed or endorsed by the publisher.
