# Supplementary material for: Crossing the Line: Seroprevalence and Risk Factors for Transboundary Animal Diseases Along the Tanzania-Zambia Border
Source: Front Vet Sci. 2022 Mar 11;9:809128. doi: 10.3389/fvets.2022.809128 (PMC8962627; doi:10.3389/fvets.2022.809128)
Supplement: Supplementary file 1 [file Table_1.docx]

**Table S1**: Predictor variables associated with animal-level seropositivity for PPRV. p-values < 0.05 are in bold. The analysis was performed on data from Tanzania due to the low seroprevalence in Zambia. n=490

| Fixed animal-level variables |  | | PPRV | | | |
| --- | --- | --- | --- | --- | --- | --- |
|  |  | Seroprevalence %  (95% confidence interval) | | Odds ratio (OR) | OR 95 % confidence interval | p-value |
| Age | ≤3 years | 0.57 (0.01-2.04) | | *Baseline* | *Baseline* | *Baseline* |
|  | >3 years | 8.70 (4.57-14.7) | | 82.0 | 2.87-2342 | **0.010** |
| Contact with wild ruminants | At least once a year | 22.2 (2.81-60.0) | | 95.1 | 1.14-7929 | **0.044** |
|  | More rarely or never | 2.49 (1.29-4.31) | | *Baseline* | *Baseline* | *Baseline* |
| Distance to the Tanzania-Zambia or Zambia-Malawi border | ≤30 km | 0.73 (0.09-2.60)  5.56 (2.90-9.50) | | *Baseline* | *Baseline* | *Baseline* |
|  | >30 km |  |  | 8.87 | 0.90-87.7 | 0.062 |
| Constant |  | |  | <0.01 | 0.00-0.02 | 0.001 |

| Random effects parameters |  | Estimate | Std. Err. | 95 % confidence interval |
| --- | --- | --- | --- | --- |
|  | District | <0.001 | <0.001 | 0 . |
|  | Village | <0.001 | <0.001 | 0 . |
|  | Household | 4.19 | 3.98 | 0.65-26.9 |

**Table S2**: Predictor variables associated with animal-level seropositivity for FMDV. p-values < 0.05 are in bold. n=931

| Fixed animal-level  variables | | FMDV | | | |
| --- | --- | --- | --- | --- | --- |
|  |  | Seroprevalence %  (95% confidence interval) | Odds ratio (OR) | OR 95 % confidence interval | p-value |
| Country | Zambia | 1.03 (0.33-2.39) | *Baseline* | *Baseline* | *Baseline* |
|  | Tanzania | 16.9 (13.7-20.5) | 17.2 | 3.32-89.6 | **0.001** |
| Age | ≤ 3 years | 5.91 (4.29-7.90) | *Baseline* | *Baseline* | *Baseline* |
|  | >3 years | 17.3 (12.9-22.5) | 5.03 | 2.46-10.3 | **<0.001** |
| Contact with cattle from other herds | At least once a month | 14.0 (11.4-17.0) | 5.40 | 0.72-40.3 | 0.100 |
|  | More rarely or never | 0.55 (0.07-1.99) | *Baseline* | *Baseline* | *Baseline* |
| Herd size sheep and goats combined | <10 | 6.38 (4.51-8.73) | *Baseline* | *Baseline* | *Baseline* |
|  | 10-20 | 9.18 (6.19-13.0) | 1.44 | 0.60-3.47 | 0.410 |
|  | 21 or more | 23.1 (15.4-32.4) | 4.75 | 1.52-14.8 | **0.007** |
| Distance to the Tanzania-Zambia or Zambia-Malawi border | ≤ 10 km | 4.24 (2.53-6.61) | *Baseline* | *Baseline* | *Baseline* |
|  | >10-30 km | 8.15 (5.18-12.1) | 1.62 | 0.55-4.74 | 0.379 |
|  | >30 km | 17.1 (12.8-22.0) | 7.96 | 1.96-32.4 | **0.004** |
| Distance to a town | ≤ 30 km | 7.87 (5.29-11.2) | 12.3 | 2.67-57.0 | **0.001** |
|  | >30-60 km | 6.09 (3.86-9.08) | 2.84 | 0.87-9.22 | 0.083 |
|  | >60 km | 14.7 (10.6-19.6) | *Baseline* | *Baseline* | *Baseline* |
| Constant |  |  | <0.01 | 0.00-0.01 | <0.001 |

| Random effects parameters |  | Estimate | Std. Err. | 95 % confidence interval |
| --- | --- | --- | --- | --- |
|  | District | <0.001 | <0.001 | 0 . |
|  | Village | <0.001 | <0.001 | 0 . |
|  | Household | 2.39 | 1.03 | 1.02-5.57 |

**Table S3**: Predictor variables associated with animal-level seropositivity for RVFV. p-values < 0.05 are in bold. n=977

| Fixed animal-level  variables | | | RVFV | | | |
| --- | --- | --- | --- | --- | --- | --- |
|  |  |  | Seroprevalence %  (95% confidence interval) | Odds ratio (OR) | OR 95 % confidence interval | p-value |
| Presence of sheep in the household | Yes | 6.00 (1.25-16.5) | | 3.26 | 0.63-16.9 | 0.160 |
|  | No | 2.59 (1.67-3.83) | | *Baseline* | *Baseline* | *Baseline* |
| Distance to the Tanzania-Zambia or Zambia-Malawi border | ≤ 10 km | 1.65 (0.66-3.36) | | *Baseline* | *Baseline* | *Baseline* |
|  | >10-30 km | 3.32 (1.53-6.21) | | 2.43 | 0.73-8.11 | 0.150 |
|  | >30 km | 3.91 (1.97-6.89) | | 2.76 | 0.86-8.85 | 0.087 |
| Constant |  |  | | 0.01 | 0.00-0.03 | <0.001 |

| Random effects parameters | |  | Estimate | Std. Err. | 95 % confidence interval |
| --- | --- | --- | --- | --- | --- |
|  | Distance | | <0.001 | <0.001 | 0 . |
|  | Village | | <0.001 | <0.001 | 0 . |
|  | Household | | 2.11 | 1.40 | 0.58-7.71 |

**Table S4**: Predictor variables associated with animal-level seropositivity for *Brucella* spp. p-values < 0.05 are in bold. n=665

| Fixed animal-level  variables | | *Brucella* spp. | | | | |
| --- | --- | --- | --- | --- | --- | --- |
|  |  | Seroprevalence %  (95% confidence interval) | | Odds ratio (OR) | OR 95 % confidence interval | p-value |
| Country | Zambia | | 1.65 (0.71-3.22) | *Baseline* | *Baseline* | *Baseline* |
|  | Tanzania | | 20.0 (14.5-26.5) | 45.8 | 10.1-207 | **<0.001** |
| Having introduced new sheep or goats to the herd | This year | | 3.23 (1.06-7.37) | *Baseline* | *Baseline* | *Baseline* |
|  | More than one year ago | | 7.80 (5.63-10.5) | 3.19 | 0.79-12.9 | 0.104 |
| Presence of community members selling small ruminants to other countries | Yes | | 2.86 (0.59-8.12) | *Baseline* | *Baseline* | *Baseline* |
|  | No | | 7.28 (5.28-9.75) | 5.40 | 0.65-44.9 | 0.119 |
| Distance to a town | ≤ 30 km | | 8.12 (5.32-11.7) | 6.04 | 1.03-35.5 | **0.047** |
|  | >30-60 km | | 3.81 (1.76-7.11) | 1.25 | 0.23-6.95 | 0.796 |
|  | >60 km | | 8.66 (4.40-15.0) | *Baseline* | *Baseline* | *Baseline* |
| Distance to the Tanzania-Zambia or Zambia-Malawi border | ≤ 10 km | | 4.27 (2.35-7.06) | 1.68 | 0.20-14.5 | 0.635 |
|  | >10-30 km | | 11.9 (7.94-17.0) | 3.82 | 0.63-23.1 | 0.144 |
|  | >30 km | | 4.00 (1.31-9.09) | *Baseline* | *Baseline* | *Baseline* |
| Constant |  | |  | <0.01 | 0.00-0.01 | <0.001 |

| Random effects parameters | |  | Estimate | Std. Err. | 95 % confidence interval |
| --- | --- | --- | --- | --- | --- |
|  | Distance | | <0.001 | <0.001 | 0 . |
|  | Village | | <0.001 | <0.001 | 0 . |
|  | Household | | 2.26 | 1.50 | 0.61-8.34 |

**Table S5**: Predictor variables associated with animal-level seropositivity for PPRV with p-values <0.25 in univariable analysis, that were later included in the multivariable models. Having introduced small ruminants to the herd, having sold sheep and goats, and the distance to the Tanzania-Zambia or Zambia-Malawi border, the Tan-Zam highway and the nearest town are also included as they were always included in the multivariable model.

| PPRV | |
| --- | --- |
| Predictor variable | p-value |
| Age | <0.001 |
| Contact with wild ruminants | 0.024 |
| Buying sheep and goats from traders or at markets | 0.044 |
| Herd size sheep and goats combined | 0.013 |
| Having introduced new sheep or goats to the herd | 0.744 |
| When the household last sold sheep or goats | 0.173 |
| Distance to the Tanzania-Zambia or Zambia-Malawi border | 0.003 |
| Distance to the Tan-Zam highway | 0.321 |
| Distance to a town | 0.297 |

**Table S6**: Predictor variables associated with herd-level seropositivity for PPRV with p-values <0.25 in univariable analysis, that were later included in the multivariable models. Having introduced small ruminants to the herd, having sold sheep and goats, and the distance to the Tanzania-Zambia or Zambia-Malawi border, the Tan-Zam highway and the nearest town are also included as they were always included in the multivariable model.

| PPRV | |
| --- | --- |
| Predictor variable | p-value |
| Contact with wild ruminants | 0.014 |
| Herd size sheep and goats combined | 0.139 |
| Having introduced new sheep or goats to the herd | 1.000 |
| When the household last sold sheep or goats | 0.374 |
| Distance to the Tanzania-Zambia or Zambia-Malawi border | 0.006 |
| Distance to the Tan-Zam highway | 0.467 |
| Distance to a town | 0.506 |

**Table S7**: Predictor variables associated with animal-level seropositivity for FMDV with p-values <0.25 in univariable analysis, that were later included in the multivariable models. Having introduced small ruminants to the herd, having sold sheep and goats, and the distance to the Tanzania-Zambia or Zambia-Malawi border, the Tan-Zam highway and the nearest town are also included as they were always included in the multivariable model.

| FMDV | |
| --- | --- |
| Predictor variable | p-value |
| Country | <0.001 |
| Age | <0.001 |
| Sex | 0.038 |
| Contact with sheep and goats from other herds | <0.001 |
| Contact with cattle from other herds | <0.001 |
| Buying from traders or at markets | <0.001 |
| Herd size sheep and goats combined | <0.001 |
| Presence of sheep in the household | 0.023 |
| Having introduced new sheep or goats to the herd | 0.265 |
| When the household last sold sheep or goats | 0.407 |
| Distance to the Tanzania-Zambia or Zambia-Malawi border | <0.001 |
| Distance to the Tan-Zam highway | 0.038 |
| Distance to a town | 0.001 |

**Table S8**: Predictor variables associated with herd-level seropositivity for FMDV with p-values <0.25 in univariable analysis, that were later included in the multivariable models. Having introduced small ruminants to the herd, having sold sheep and goats, and the distance to the Tanzania-Zambia or Zambia-Malawi border, the Tan-Zam highway and the nearest town are also included as they were always included in the multivariable model.

| FMDV | |
| --- | --- |
| Predictor variable | p-value |
| Country | <0.001 |
| Contact with sheep and goats from other herds | 0.006 |
| Contact with cattle from other herds | <0.001 |
| Buying from traders or at markets | <0.001 |
| Herd size sheep and goats combined | 0.003 |
| Presence of sheep in the household | 0.046 |
| Having introduced new sheep or goats to the herd | 0.790 |
| When the household last sold sheep or goats | 0.029 |
| Distance to the Tanzania-Zambia or Zambia-Malawi border | <0.001 |
| Distance to the Tan-Zam highway | 0.036 |
| Distance to a town | 0.097 |

**Table S9**: Predictor variables associated with animal-level seropositivity for RVFV with p-values <0.25 in univariable analysis, that were later included in the multivariable models. Having introduced small ruminants to the herd, having sold sheep and goats, and the distance to the Tanzania-Zambia or Zambia-Malawi border, the Tan-Zam highway and the nearest town are also included as they were always included in the multivariable model.

| RVFV | |
| --- | --- |
| Predictor variable | p-value |
| Buying from traders or at markets | 0.156 |
| Presence of sheep in the household | 0.156 |
| Having introduced new sheep or goats to the herd | 0.816 |
| When the household last sold sheep or goats | 0.747 |
| Distance to the Tanzania-Zambia or Zambia-Malawi border | 0.141 |
| Distance to the Tan-Zam highway | 1.000 |
| Distance to a town | 0.120 |

**Table S10**: Predictor variables associated with herd-level seropositivity for RVFV with p-values <0.25 in univariable analysis, that were later included in the multivariable models. Having introduced small ruminants to the herd, having sold sheep and goats, and the distance to the Tanzania-Zambia or Zambia-Malawi border, the Tan-Zam highway and the nearest town are also included as they were always included in the multivariable model.

| RVFV | |
| --- | --- |
| Predictor variable | p-value |
| Buying from traders or at markets | 0.108 |
| Presence of sheep in the household | 0.105 |
| Herd size sheep and goats combined | 0.095 |
| Having introduced new sheep or goats to the herd | 0.888 |
| When the household last sold sheep or goats | 0.855 |
| Distance to the Tanzania-Zambia or Zambia-Malawi border | 0.136 |
| Distance to the Tan-Zam highway | 0.906 |
| Distance to a town | 0.288 |

**Table S11**: Predictor variables associated with animal-level seropositivity for *Brucella* spp., with p-values <0.25 in univariable analysis, that were later included in the multivariable models. Having introduced small ruminants to the herd, having sold sheep and goats, and the distance to the Tanzania-Zambia or Zambia-Malawi border, the Tan-Zam highway and the nearest town are also included as they were always included in the multivariable model.

| *Brucella* spp. | |
| --- | --- |
| Predictor variable | p-value |
| Country | <0.001 |
| Sex | 0.121 |
| Contact with sheep and goats from other herds | 0.063 |
| Contact with cattle from other herds | <0.001 |
| Presence of community members selling small ruminants to other countries | 0.131 |
| Having introduced new sheep or goats to the herd | 0.047 |
| When the household last sold sheep or goats | 0.018 |
| Distance to the Tanzania-Zambia or Zambia-Malawi border | 0.001 |
| Distance to the Tan-Zam highway | 0.002 |
| Distance to a town | 0.086 |

**Table S11**: Predictor variables associated with herd-level seropositivity for *Brucella* spp., with p-values <0.25 in univariable analysis, that were later included in the multivariable models. Having introduced small ruminants to the herd, having sold sheep and goats, and the distance to the Tanzania-Zambia or Zambia-Malawi border, the Tan-Zam highway and the nearest town are also included as they were always included in the multivariable model.

| *Brucella* spp. | |
| --- | --- |
| Predictor variable | p-value |
| Country | <0.001 |
| Contact with cattle from other herds | <0.001 |
| Presence of community members selling small ruminants to other countries | 0.183 |
| Having introduced new sheep or goats to the herd | 0.067 |
| When the household last sold sheep or goats | 0.136 |
| Distance to the Tanzania-Zambia or Zambia-Malawi border | 0.033 |
| Distance to the Tan-Zam highway | 0.227 |
| Distance to a town | 0.158 |
